# Learning Processes and Trajectories for the Reduction of Antibiotic Use in Pig Farming: A Qualitative Approach

**DOI:** 10.3390/antibiotics4040435

**Published:** 2015-10-22

**Authors:** Nicolas Fortané, Florence Bonnet-Beaugrand, Anne Hémonic, Carole Samedi, Arnaud Savy, Catherine Belloc

**Affiliations:** 1 Institut National de la Recherche Agronomique (INRA), UR 1323 RiTME, 65 boulevard de Brandebourg, F-94205 Ivry-sur-Seine, France; E-Mail: arnaud.savy@ivry.inra.fr; 2 LUNAM Université, Oniris, Ecole nationale vétérinaire, agroalimentaire et de l’alimentation Nantes-Atlantique, UMR Biologie, Epidémiologie et Analyse de Risque en santé animale, CS 40706, F-44307 Nantes, France; E-Mails: florence.beaugrand@oniris-nantes.fr (F.B.-B.); carole.samedi@oniris-nantes.fr (C.S.); catherine.belloc@oniris-nantes.fr (C.B.); 3 Institut National de la Recherche Agronomique (INRA), UMR1300, F-44307 Nantes, France; 4 IFIP-Institut du porc, Domaine de la Motte au Vicomte, BP 35104, F-35651 Le Rheu, France; E-Mail: anne.hemonic@ifip.asso.fr

**Keywords:** antibiotics, animal health, farmers, veterinarians, qualitative approach, learning process, trajectory of change

## Abstract

Since 2011, French public policy has been encouraging a reduction in the use of antibiotics in animal farming. The aim of this article is to look at how some farms have already managed to lower their consumption of antibiotics, and to highlight the levers of change in farming health practices. Our research uses a qualitative study based on 21 semi-structured interviews with farmers and veterinarians in the French pig-farming sector. We use the notion of “trajectory of change” to examine, over time, the intersection of the technical, economic, social and organisational determinants which affect the reduced use of antibiotics. The “learning process” concept makes it possible to take account of the way in which the actors assimilate, appropriate and implement new health practices. We have identified three interdependent levels of learning: technical learning, cognitive learning and organisational learning.

## 1. Introduction

The fight against antibiotic resistance is a subject of much discussion in Europe. Over recent years, a certain number of measures have been introduced to encourage a reduction in antibiotic use. In 2001, the European Union developed a programme in the fields of monitoring, research, prevention and international cooperation in human medicine. For veterinary medicine, it has gradually introduced the monitoring of antimicrobial resistance to zoonotic bacteria and of the use of antimicrobial products in animals. In 2006, the EU voted a ban on the use of antimicrobial products as growth promoters in animal feed. In 2011, resistance to antibiotics was the theme of World Health Day, reconfirming the determination to come up with a global plan of action. In 2011, the French minister of agriculture introduced the “Ecoantibio” plan, which mobilised the entire range of animal health actors and targeted a 25% reduction in veterinary use of antibiotics over the next five years. This objective is nevertheless only feasible if we have a proper understanding of the factors that determine the use of antibiotics in animal farming.

Several expert reports have provided a considerable amount of quantitative information on the evolution in resistance to pathogenic bacteria [1], on annual antibiotic sales [2] and on the molecules used, depending on the diseases or animals being treated [3]. For example, between 2008 and 2013 in France, the animal exposure to antibiotics has decreased by 15.7% and the total amount of antibiotics sold has diminished by 30.7%. Other studies have examined the question of the dosages used, depending on the type of treatment given on farms [4] or the global cost of antimicrobial use in livestock [5]. These quantitative data are now starting to be completed with more precise descriptions of use and with the perceptions and motivations behind the use of antibiotics. Most of this work comes from veterinary research and use qualitative approaches (essentially based on behavioural theories) either to understand the discrepancy between political and scientific directives and farmer practices [6], or to highlight farmers’ perceptions, habits and motivations when they use antibiotics or other alternatives [7,8,9,10,11]. More often than not, these studies conclude that farmers receive insufficient information on the proper use of antibiotics [12,13,14]. Even though veterinarians have to handle social pressure that can influence their prescription habits [8,15], they are thus put forward as a “solution”, *i.e.*, as actors who can instill “awareness” and who are able to introduce the pedagogy and communication needed for practices to change [11,16]. Whilst we believe this approach to reduced antibiotic use to be pertinent, it does not explain how actors’ perceptions, motivations and attitudes are constructed, rely on their professional experience and can evolve. These questions therefore merit further discussion by mobilising works in the social sciences and by creating a truly interdisciplinary approach.

Although animal health issues have not been examined in many interdisciplinary works, numerous studies on crop production are nevertheless available. They look at processes of agricultural change, particularly with regard to the reduced use of phytosanitary products or conversion to organic farming. They bring agronomists, ergonomists and sociologists together to consider approaches in terms of “trajectories” (*i.e.*, the long-term evolution of farming practices) and to analyse the way in which farmers organise their transition to a different type of agricultural production. The notion of trajectory, based on qualitatively analysed career narratives, allows us to challenge uses from a longitudinal perspective—the only way to understand how levers of change can (or not) be activated [17,18,19]. On the one hand, via the notion of “technical itinerary” [20], these works bring to light the technical and organisational factors affecting changes in practice. On the other hand, they highlight learning devices [21,22] and the role of socio-professional networks in the trajectory of change [23]. Overall, this research shows that the withdrawal or reduction of certain agricultural inputs takes place at multiple levels and that there are different ways of modifying one’s practices in order to achieve reduction objectives.

In this article, we intersect these two groups of research (perceptions and motivations on the one hand, trajectories on the other) and analyse the “learning processes” which have allowed farmers to reduce their use of antibiotics. The study looks at French pig farmers and was conducted as part of the interdisciplinary TRAJ project (Trajectories of change in the use of antibiotics in animal farming), financed by the Institut National de la Recherche Agronomique (INRA’s) GISA meta-programme (Integrated Animal Health Management (Gestion Intégrée de la Santé Animale)). The central hypothesis that the TRAJ project is attempting to document through qualitative studies is twofold: firstly, that a change in farming practices is based not only on technical and economic factors, but also on social and organisational elements; secondly, that change is not the responsibility of any single actor—in this case the farmer—but of the relationship system that the latter weaves with his/her technical and health advisors.

## 2. Methods

Our study took place in the intensive pig-farming sector in the west of France, essentially in the Côtes d’Armor region. This sector was one of the largest users of antibiotics, but its consumption has considerably decreased since 2010, when pig producers professional organizations have set up a moratorium on the preventive use of last generation antibiotics (especially cephalosporins and fluoroquinolones). The exposure to C3G and C4G has therefore diminished by 66% between 2010 and 2013 [2]. Nevertheless, little is known on the way pig farmers succeed in reaching this objective. Our study on the trajectories of antibiotics reduction shed some light upon these processes.

In keeping with the qualitative approach, we carried out semi-structured interviews in order to grasp the reasons behind animal farming practices and the subjective significance that individuals attach to their attitudes [24]. In total, we carried out 21 interviews between April and June 2014, with an average length of two hours: 11 farmers (members of different cooperatives, *i.e.*, producer organizations) and their 10 veterinarians (two farmers had the same veterinarian). This sample was built through the intermediary of the Institut Technique de la Filière Porcine (Technical institute for the pig-farming sector) (IFIP), which is one of the project partners. IFIP possesses technical and economic data on the sector and was able to classify the farms according to their veterinary costs and to identify those whose expenditure on antibiotics has dropped the most over the last decade. IFIP sent us a list of the 20 farms in the west of France, which had recorded the most significant reductions in antibiotic use. Following an initial contact through their veterinarians, seven farmers agreed to take part in the study. A second list provided by IFIP (showing the next twenty farms) allowed us to recruit four more farmers and thus reach the recommended empirical saturation point for qualitative study sampling. This threshold, generally defined as being between 10 and 12 interviews per actor category, represents the limit above which the researcher will obtain no additional information relating to the object of his/her research [25,26]. Further interviews were carried out with technicians from the cooperatives in order to support certain aspects of our analysis, but we have not used them in this article. All of the interviews complied with INRA’s Ethics Charter, which guarantees the anonymity and confidentiality of the data provided by participants. The interview grid was built around three axes:

- Context: history of the farm or of the veterinary practice, interviewee’s career, farm operation and relations with other actors in the sector (particularly cooperatives).
- Use of antibiotics (and/or of alternatives): farmers’ relations with their technical and health advisors, decision-making factors and motivations regarding the various treatments used, organisation of farm work.
- The demedication process (*i.e.*, reduction in antibiotic use): developing strategies to reduce use, technical/economic effects and consequences, development of new tools and of the devices required to change practices.

The interviews were recorded on a Dictaphone and transcribed in full. They were then analysed using a thematic grid on Sonal^®^ software. The analysis grid contained four major themes relating to the four sets of factors affecting the use of antibiotics in animal farming:

- Technical and health factors which come into play in the prescription and use of veterinary medicines and in the implementation of health and biosafety measures.
- Economic factors relating to the costs, income and profit margins of the farms and the veterinary practices, particularly those relating to the price of antibiotics and sundry inputs (notably feed and alternatives).
- Social factors, relating to the pathways of the actors interviewed, their perception of health risks, their beliefs concerning the question of antibiotic resistance, the future of the sector, the constraints and motivations when beginning a demedication process.
- Organisational factors, concerning relations between individual actors (farmers, veterinarians and, to a lesser extent in this case, technicians) and groups of actors (the operation and strategies of the cooperatives, veterinary practices and farms), and the division of health-related work on the farm.

## 3. Results and Discussion

### 3.1. Raw Data Extracted from the Interviews

The following tables set out the main data extracted from the interviews. Table 1 highlights the principal factual elements that the actors believe to be important in their trajectory of change. For the most part, the technical, economic and health determinants appear in the “building” and “farming practice” columns. The social and organisational determinants are found in the “set-up and training”, “manpower, workshops” and “professional networks” columns. The “significant events” column shows the points in time which, in the opinion of the interviewees, constituted important stages in their antibiotic reduction trajectories (either in terms of decision-making, or with regard to effective implementation of a change in practice). Table 2 presents the social characteristics and the professional practices of the veterinarians. As generally happens with qualitative studies based on semi-structured interviews, the information gathered is more disparate than would be the case with information collected through questionnaires, but it allows us to more accurately trace the variability in trajectories of change, which take place over long periods and whose different factors can combine in a highly diverse manner.

**Table 1:** Main aspects of trajectories of change in the 11 farms studied.

Farm, Cooperative, French *Département*	Set-up Year and Farmer Training	Manpower, Herd Size and Other Farm Enterprises	Building Evolution	Type of Piggery and General Management	Disease Control	Changes in Socio-Professional Network	Significant Events
Farm 1 Coop A Dép 72	1989: takeover of a family farm	1 pers. 80 sows. Grain production	1998: Creation of a farmer-fattener building	Metering pump Blank feed Hay in gestation building.	Sow vaccination. Systematic disinfection.	1996: Change of cooperative in order to remain with a technician	Problem with feed. Veterinarian advice (metering pump and vaccination strategy).
Farm 2 Coop B Dép 72	1980	2 pers. 150 sows. Ewe farming until 1987.	Gradual renovation. 2013: free-range sows.	Weaning at 21 days	2008: Circovirus and mycoplasma vaccination + feed supplementation (Systematic preventise use of antibiotics (added to piglets feed).) ceased	1997: Change of cooperative and veterinarian. Then 5 or 6 changes of veterinarian.	Recurring problems of ileitis post-weaning.
Farm 3 Coop B Dép 22	1994: Takeover of a family farm, agricultural training.	3 pers. 310 sows. Milk production.	Renovation: 1991: fattening building 1992: Gestation building 1996: Maternity building	Metering pump	2011: Supplementation ceased for early age. PRRS (Porcine productive and respiratory syndrome.) and mycoplasma vaccination.	1996: Change of cooperative for feed	2000: PMWS (Post-weaning multisystemic wasting syndrome.)
Farm 4 Coop B Dép 22	1984: Agricultural training, pig-farming course.	2 pers. 150 sows, Hotel business.	Gradual, but substantial investment.	1992: Multiplication ceased. Weaning at 21 days	1992: Supplementation ceased.	End 1990s: Change of cooperative. Then 3 changes of veterinarian	1992: PMWS. 2013: Compliance with “well-being” standards (This standard requires a group housing (rather than confinement) for pregnant sows. Farmers and vets generally consider that this change in housing practice was an opportunity to change other aspects of farming practices, such as animal health management.). Ethical dilemmas relating to hotel customers (The farmer’s wife manages a little hotel near the farm. Some of their clients were worried about the antibiotics used in the piggery and their possible exposure to resistant bacteria. The farmer and his wife consider that it was an additional motivation to reduce antibiotic use.)
Farm 5 Coop A Dép 53	2004: Takeover of a family farm	2 pers. 150 sows.Grain production.	1984: Straw in fattening building (400 places) 2004: Slatted floors in fattening building (600 places) 2008: Slatted floors in all buildings.	2009: Farm makes its own feed; Weaning at 28 days; Metering pump	2011: Supplementation ceased + circovirus, mycoplasma, ileitis and flu’ vaccinations.	2009: Met a sales engineer who advised him to make his own feed.	Sows with respiratory problem. Vaccination strategy implemented with veterinarian.
Farm 6 Coop B Dép 53	1996: Takeover of a family farm. Agricultural training	2 pers. 85 sows. Milk production.	2002: complete renovation. 2006: ventilation system changed.		2002: Supplementation ceased. 2006: tylosin removed. 2012: complete cessation of antibiotics. Circovirus, mycoplasma and PRRS vaccination.	2002: Change of cooperative.	2000: reproduction problem. 2004: PRRS. 2006: Coughing problem post weaning.
Farm 7 Coop B Dép 22	1991: Takeover of a family farm, Vocational training certificate (BEP) in agriculture	2 pers. 250 sows.	Gradual until 2005. 2006: fattening. 2008: slurry treatment plant.	1992: installation of a metering pump. Weaning at 21 days.	Mycoplasma vaccination. 2007:Supplementation ceased.	1992: change of feed cooperative; 2nd vet for feed. Veterinarian then changed twice.	2010: Compliance with “well-being” standards. Problems with ileitis.
Farm 8 Coop B Dép 22	2009: Takeover of a family farm. BEP, Bac pro, BTS	3 pers. 400 sows.	2009: Complete renovation with installation of air filtering system.	Weaning at 21 days.	PCV (Pneumococcal Conjugate Vaccine.) vaccination. Gradual cessation of supplementation between 2009 and 2014.	2014: change of cooperative.	1998: PMWS followed by restocking of herd. Compliance with “well-being” standards.
Farm 9 Coop C Dép 22	2002: Trained in sales (3 years as feed technician).	1 pers. 130 sows. Milk and grain production.	2009: complete renovation of maternity building	Weaning at 21 days.	Circovirus vaccination. Supplementation ceased.	Veterinarian changed on several occasions. Change of technician in 2010.	Forgot to stock up on antibiotics … and no problems.
Farm 10 Coop B Dép 44	1988, Vocational training certificate (BEP) in agriculture, specialisation certificate in pig production.	5 pers. 450 sows.	2001: renovation of fattening building. 2005: fattening building enlarged + post-weaning building renovated.	Metering pump	PCV and mycoplasma vaccination. Supplementation ceased.	2009: change of cooperative. Change of veterinarian	2005: PMWS and PRRS, followed by implementation of a new vaccination strategy
Farm 11 Coop B Dép 22	2000: Takeover of a family farm, BTS Technico-commercial.	2 pers. 330 sows.	2012: complete renovation of all buildings.		2012: introduction of disinfection periods between litters.	2002: Change of feed cooperative.	PRRS and PMWS problem in the early 2000s. 2012: compliance with “well-being” standards.

**Table 2:** Veterinarians’ characteristics and professional practices.

Vets (Age and Sex)	Experience in Pig Sector (Number of Years, Choice and Motivation)	Previous Professional Activities	Current Position (Vet Office, Number of Vets in This Office)	Number of Farms in Charge	Professional Relationship with Technicians	Demedication Strategy (of the Vet Office or the Cooperative)
Vet of farm 1 36, M	4 years (by choice after an experience in Romania)	Vet for two farms of 2500 sows, for 4 years	A (for 4 years) Less than 5 vets	60–70	Works in tandem, in order to improve support’s efficiency	Antimicrobial consumption indicator (+communication on the results)
Vet of farm 2 36, M	2 years (by opportunity, wanted to work in a technical sector)	Mixed practice for 9 years	B (for 9 months) More than 20 vets	130	Thinks they have complementary skills but need to be better defined	Audit of demedication
Vet of farm 3 57, M	2 years (occasional replacement, pre-retirement)	Practice in dairy production, small animals medicine and surgery, pharmaceutical industry	B (for 1 year) More than 20 vets	200	Thinks it is important to help farmer’s decision-making	Audit of demedication
Vet of farm 4 (same as farm 8) 35, M	5 years (by opportunity)	Mixed practice for 6 years, veal calf production for 3 years	B (for 4 years) More than 20 vets	100	Doesn’t work in tandem	Audit of demedication
Vet of farm 5 49, F	22 years (by choice, real passion for the pig production)	Pharmaceutical industry for the pig sector	A (for 10 years) Less than 5 vets	15 (+animal feed factories)	Works in tandem for preventive measures (but not for curative ones)	Antimicrobial consumption indicator (+communication on the results)
Vet of farm 6 37, F	10 years (by opportunity, wanted to work in a technical production)	Mixed practice for 2 years	B (for 8 years) More than 20 vets	120	Doesn’t work in tandem	Audit of demedication
Vet of farm 7 45, M	16 years (by opportunity, wanted to work in a technical production)	Mixed practice for 2 years	B (14 years) More than 20 vets	120	Variable (depending on their availability)	Audit of demedication
Vet of farm 8 (same as farm 4) 35, M	5 years (by opportunity)	Mixed practice for 6 years, veal calf production for 3 years	B (for 4 years) More than 20 vets	100	Doesn’t work in tandem	Audit of demedication
Vet of farm 9 31, M	7 years (by choice, real passion for the pig production)	none	C (for 6 years) Less than 10 vets	100	Doesn’t work in tandem	Developed a new feed (without antibiotics) to replace supplemented feed
Vet of farm 10 45, M	18 years (by opportunity, wanted to work in a technical production)		B (for 5 years) More than 20 vets	100	Thinks it is important to work together	Audit of demedication
Vet of farm 11 37, F	12 years (by opportunity, likes the pig production and wanted to be employee rather than independant	Pig sector (employee of a cooperative for 5 years, then indepndant for 4 years)	B (for 3 years) More than 20 vets	100	Insists on the importance of trust to make the relationship efficient	Audit of demedication

The decision to reduce the use of antibiotics can therefore be based on a significant health event (farms 2, 5, 6, 8, 10 and 11), on the introduction of new specifications involving a new economic and health strategy (farms 3, 4, 7, 8 and 11), on ethical considerations relating to the consequences of using antibiotics (farm 4), or even on nothing at all—in other words, antibiotic use may have dropped without this being a specific objective (farm 9). To this may be added, for almost all farmers interviewed, technical, economic and health factors such as the renovation of buildings or vaccination strategies, and social or organisational factors such as a change in the professional network (change of cooperative or the arrival of a new veterinarian, leading to new practices). The importance of the socio-professional network is quite difficult to objectify but every farmers explained that their change of practice were discussed and negotiate with their technical and health advisers. Besides, some farmers deliberately changed their veterinarian or their cooperative in order to change farming practices and decrease their antibiotic consumption (farms 1, 6, 8 and 10). Interviews with veterinarians confirm this point: almost all of them (except farms 4/8 and 9) consider that working closely with the technician (and of course the farmer) is important, especially to define and implement demedication strategies. This point of view is even stronger when veterinarians chose to work in the pig sector for this kind of professional relationships, *i.e.*, work in tandem with technician, not being limited to sanitary issues but integrate animal health with technical (such as feed and housing) and economic ones. Finally, we must also take into consideration the fact that the question of economic performance was regularly raised during the interviews. This is presented as an important factor in as much as profit margins need to be maintained (or improved) in order for the change to be sustainable. The table therefore highlights the diversity of factors that affect a reduction in the use of antibiotics, and consequently the variety of trajectories of change (relating to the use of antibiotics in animal farming) that can exist. We will now enlight and interpret these data by using several concepts of the above-mentioned literature (especially the notion of “learning process”), in accordance with the qualitative research methodology [27].

### 3.2. Qualitative Analysis of the Data

The question of change and of beginning the process of reducing the use of antibiotics is therefore anchored in diverse trajectories which take place over long periods and which are structured by farmers’ experiences. Changes in agricultural practices (in this case health practices in animal farming) cannot therefore be considered as fully intentional ruptures, but rather as the mobilisation and exploration of new ways of doing things and as the definition of new production criteria [21]. It requires new forms of learning that cross-reference and intersect at different levels with the four factors being studied. We have identified three “learning processes” which are deployed in each trajectory of change [28,29]. These processes show us that actors’ perceptions, motivations and attitudes must be grasped from an evolutive perspective and, above all, that they are linked to work practices and modes of organisation which also evolve. Besides, learning processes show us how farmers face difficulties and acquire new skills and abilities in order to reach their objective of reducing antibiotics. At the end of the day, trajectories of change relating to the use of antibiotics in animal farming can only be understood in terms of the diverse factors that comprise them, and of the way in which the latter intersect in specific learning processes.

#### 3.2.1. Technical Learning

This process involves learning to use new animal health management tools. It might be a case of implementing a new health strategy, a new farming practice or technical modifications to a building. The introduction of a new technical tool for farm management, with a view to reducing one’s antibiotic consumption, requires one to understand new methods and new information in order to put the different functions to the best possible use and to make the most of the advantages one hopes to gain. The notion of technical learning therefore relates to an initial lever for a change in practice which, whilst it may seem obvious, is not always self-evident. Many of the farmers we interviewed explained that in order for new farming techniques or tools to have any real effect on their use of antibiotics, they had had to “learn” to “use them properly” and to integrate them with the other technical devices on their farms.

The installation of a metering pump, which allows one to provide a metaphylactic treatment through drinking water when there is a health problem, is a perfect example of this learning process and of the difficulties that go with it. This piece of equipment has allowed all of the farmers we interviewed to stop the systematic supplementation of antibiotics in piglet feed and to only use such medicines when they are necessary (curative or metaphylactic use rather than preventive use). However, the use of a metering pump requires a certain number of technical learning processes to ensure that it works properly and to guarantee the desired effects on overall antibiotic consumption. The farmer must be able to calculate the required dosage (posology), know when to give the treatment and check that the drugs have been homogeneously consumed by the herd as a whole. However, according to veterinarians, farmers do not always find it easy to learn this process:

“There are typologies of farmers where we can say that we can stop, that they’ll be able to use the metering pump, and then there are typologies of farmers where we really aren’t sure that they’re going to be able to manage with the metering pump. Either because they don’t go to see their pigs enough to be able to rapidly detect the first signs, or because as far as they are concerned, the metering pump may as well be a nuclear power plant. Even if we train them or whatever, we know they aren’t rigorous, that they’ll never be able to calculate the correct doses to be added. It’s just too complicated for them. We’re seeing fewer and fewer cases like that, but there’s still a somewhat elderly generation in practice”;

[Veterinarian]

“A metering pump means a few extra constraints, because before that it wasn’t hard to have a lorry load of medicated feed and pour it into the silo, and then there was the distribution chain to feed the animals, there was no extra work to do. But now you have to put the drug in the metering pump, check that it’s the right dose, the right quantity, so you have to do a bit of calculating. Check that the flow is correct, check that it’s going to the right place, that it’s properly absorbed, clean the metering pump again, do the maintenance, so there’s a little bit of extra work to do.”

[Veterinarian]

Metering pumps are an excellent example of how a technical tool allows one to reduce the use of antibiotics in pig farming. More than half of interviewed farmers declared that it changed their attitude towards their antibiotic consumption, by making it visible: when the feed is no longer automatically supplemented and you have to administer the pharmaceutical inputs yourself, you are necessarily aware of their presence and you can measure them, objectify them and manage them. So the technical learning process is not a simple practical training course but a real transformation of the ways of looking at and performing animal health management. However, it is only one of the aspects of changing practice. It must go hand in hand with a global modification of risk perception (cognitive learning) and with a reorganisation of work on the farm (organisational learning).

#### 3.2.2. Cognitive Learning

As far as the use of new health management tools is concerned, cognitive learning requires a recomposition of the farmers’ experiences, which are to some extent characterised—as is the case for farmers who have converted to organic farming—by a change in the relationship with risk [18]:

“No, you need to know a bit about your farm and your animals, and see how it behaved previously … if you have a really bad health situation, you know that when it starts, it won’t stop, if you’ve got a spot of bother… well, you know, you have periods, a little bit, from time to time, but you keep them under control, if you’ve got a vaccination already set up for certain ones … mycoplasmas for example, PRRS or whatever, you know that you can allow yourself some time to think. You don’t have to immediately get cracking.”

[Farmer]

What we find here regarding the construction of experience in relation to a vaccine is what we have already seen in the previous example, *i.e.*, learning processes which develop as one uses a metering pump. Whilst it is still vital to monitor the animals, this must now be done in other ways: although farmers involved in intensive pig farming learned long ago how to spot clinical signs at herd level (rather than in individual animals), they now also need to distinguish between times when only a few animals need to be treated (curative treatment, usually by injection) and times when it is preferable to treat an entire group of animals (metaphylactic treatment using the metering pump):

“How do you decide whether to treat? How do you know? It’s easy to see if a piglet is ill. A piglet will isolate itself, it won’t drink, its eyes will be somewhat hollow, maybe with a bit of swelling. And it’ll lie down. So if you’ve just got one case, or two or three … but then after a couple of days it might get out of control. If we can cope with two or three cases, no problem, we’ll leave the others, I’m not going to treat the whole herd. But if I see that things are going downhill … And it’s always ten days after weaning. I wean at 21 days, the weekend, ten days later you need to be very vigilant. If there’s the slightest sign, we might decide to use the metering pump. So up to three cases, you treat them individually? Yes, yes. And beyond that? We’ll treat the whole lot.”

[Farmer]

Works on the transition process in organic farming have shown that a farmer’s experience is essentially built and adjusted through experimentation, related to the introduction of a new technique [17]. A change in the way antibiotics are used in animal farming involves similar dynamics; in other words, the learning processes are shaped over a relatively long period of time. The farmers we met often feel that they are still in a learning phase, even if they began their demedication approach several months or years ago. In any case, it is an illusion to think that a practice changes at a given point in time and that one can accurately distinguish between the before and the after. On the contrary, change is a process, it runs alongside the (technical) experiments that are needed to modify the subjective experience, *i.e.*, the actor’s attitudes.

“Your veterinarian told me that for you, and even for others, it’s really a case of “trial and error, trying things out, going back a step, etc.” Does that mean you test one litter, one batch, before you decide to go ahead with everything? In general, when we do something, we give ourselves 4 or 5 months to wait for the results, to see if what we’ve done is successful or not. You can’t tell just from one or two litters. You need almost 4 or 5 months to get the answers, especially with respiratory problems. When it’s the digestive system, that’s different, because you get an almost immediate answer, but for respiratory issues it’s a long-term thing. The problem is that with pigs, when you change something, there are always other litters present, which didn’t get the treatment, so the old method or the previous situation that existed before is still ongoing. To find out what is really happening, you pretty much need to wait until all the animals in the building are either healthy or having the treatment. Then you need to be able to measure it, to have the results in figures. But generally speaking the figures confirm what we’ve been seeing every day. We farmers see our animals every day, we don’t need to wait for the figures to know whether things are alright or not.”

[Farmer]

We can clearly see the extent to which modifying an aspect of technical animal health management, with a view to reducing the use of antibiotics, is worthless without the parallel adaptation of knowledge which will not only allow the new tool to work properly, but also make it possible to assess its effects on the farm’s results. However, such a modification to ways of perceiving animal health—or, more broadly, the problem of antibmicrobial resistance—only becomes possible if it goes hand in hand with a modification to ways of working and hence of organising the activities of people who carry out work on the farm.

#### 3.2.3. Organisational Learning

There are three fundamental aspects to the organisation of work on a livestock farm: the organisation of labour, equipment and building layout, and farming methods [20]. These three elements are therefore also modified during the demedication period, in as much as the technical and cognitive learning processes—*i.e.*, the acquisition of new skills and knowledge—are based on a transformation of work methods [30]. This intersection between the different processes can be seen, for example, when using monitoring tools to assess a farm’s technical and health performances. These tools encourage us to think differently about animal health and offer a new perception of the problems that farmers face on a daily basis; to be effective, work must be organised in a way that suits their use:

We record losses. We record prolificacy, productivity, growth. Every fortnight we pick up the pigs [to take them to the slaughterhouse], so we see what the slaughter weight is. We’ve got what’s written on the slaughterhouse forms, so these are criteria that we see every day. We have the muscle content. When you get low levels of muscle content, when the pigs aren’t well, generally you can see that for yourself. All of this helps us on a daily basis to see what’s going on. We see our animals two or three times a day, so we have to react before a criterion comes to light. The important thing is to react fast.

[Farmer]

From the farmer’s standpoint, work management means time management. Day to day or week to week, the division of tasks and activities is of course affected by the strategies that are implemented to reduce the consumption of antibiotics. Replacing an antimicrobial treatment by an alternative such as vaccination (in our case, 8 farmers amongst the 11 that were interviewed) clearly gives farmers the feeling that they are being more effective in managing the health of their animals, but also, and perhaps more importantly, it means that they are organising their work differently, with more serenity. Contrary to the notion that farmers continue to use antibiotics because they are “anti-stress” or a “crutch” to maintain the farm’s economic and technical performances [12,31], farmers who are committed to demedication switch to “non-antibiotic” farming in order to achieve “carefree management”:

When did you say to yourself “health-wise I’m going to change the way I do things, I’m going to use vaccines”? It was the fact that we were always playing catch-up, we were always one step behind. When you’re playing catch-up, you can’t cope and the results are never satisfactory. When you give the pigs antibiotics, you can see that they get better, but they are never as healthy as they were before they became ill. What I wanted was to no longer have my metering pump, to no longer be giving the pigs a treatment left, right and centre. And also, afterwards you no longer know what antibiotic to use, because nothing works any more. Now, the fact that I’ve got a mycoplasma vaccination which suits my herd … (…) It takes a bit more time to do the injections, a booster shot after three weeks, but you save so much time afterwards if you have no more problems. The one hour you lost … So as far as I’m concerned, the advantage of vaccines is that everything is planned. When you’re organising your day, you know it’s all planned.

[Farmer]

Moreover, the question of work organisation is not just an independent variable that farmers must learn to manage in order to reduce their antibiotic consumption. On the contrary, demedication is sometimes first and foremost a way of rethinking one’s work methods and it is these organisational questions that determine the desire and decision to reduce antibiotic use. The reduced use of antimicrobial products can be compared to other types of change in farming practice: the modifications are not made solely for their own sake, but well and truly because they help create new ways of working and, ultimately, of being a farmer [32]. So as far as our interviewees are concerned, a health strategy choice is not based solely on its technical and economic effectiveness but also on the forms of farming work organisation that they enable:

“When you don’t have any problems, or when you just have one problem in the whole year, you’re more relaxed when going to work. Even in everyday practice, if one morning you haven’t been able to take a look at your pigs because you’ve been busy doing something else, you haven’t been able to check the feed, you haven’t seen them, instead of seeing them twice a day you’ve only seen them once, you know that there’s a 99% chance that everything is fine.”

[Farmer]

#### 3.2.4. The Importance of Social Networks in the Learning Processes

Whilst a reduction in the use of antibiotics is a medium or even long-term process that involves three levels of learning, our data highlight at least one broader condition for success. A change of farming practices is never the sole responsibility of the farmer; the entire network of actors who carry out work on the farm is concerned, particularly technical and health actors. This aspect of trajectories of change is well-documented in works on the reduced use of pesticides and the transition towards organic farming [19,23], but the case of antibiotic use is no exception [8].

Firstly, the various experiments which have been developed to allow farmers to build the experience they need for the above-mentioned learning processes mobilises actor networks within which there is a circulation of information on farming innovations:

“How do you get information on new treatments, on new health strategies, on new things relating to farming methods? Do you receive visits from veterinarians from the cooperative, from the feed manufacturer, or from other technical sales representatives? Yes, we get information like that, or from farmer colleagues who we see at meetings, or else from private sources. So by talking to people here, there and everywhere. I generally tend to trust my vet for everything to do with health. As far as technical farming methods and ways of looking after litters are concerned, we’ve known how to raise pigs for years, we know all the methods. It’s all old hat.”

[Farmer]

Secondly, how these networks influence farmers’ practices depends on the intensity of their relations with their advisors [23]. Of course, this intensity might depend on how long the relations have existed, or in certain cases on their extracurricular extension, but very often it is the learning phenomena that are constructed through the working relationship which enable it to become long-lasting and to have a decisive effect on the process of change:

“Personally, in my relations with farmers… I try to be didactic, *i.e.*, I teach them too. In other words, when I do an autopsy, if the farmer is there, I’ll say “Look, you can see this, you can see that, so it’s this, it’s that”… what I mean is I like teaching people. I want them to understand why I say this or that. Sometimes, you can say to yourself “he said that, but”, if you don’t understand the reasons and the necessities, then of course you don’t take it on board. So I try to explain all that to them. So if they’re not too daft, they understand. Among my customers there are people with a certain level… what I mean is that some of my customers are agronomists. These guys have the same level as me, they have no problems understanding all that, there’s no problem. (…) They know all about the issues that they have to deal with every day, on their farms, throughout time; they are able to recognise things just as well as you can, there’s no problem, because you’ve taught them how to do it. It’s when something new comes along, that’s when they have to call the vet.

[Veterinarian]

Finally, we must stress that socio-professional networks are not mere interpersonal relations allowing people to share knowledge; on the contrary, they often owe their robustness and concrete effects to devices for the management of farming practices, which orient actors’ decisions and which can be used as a basis for implementing their demedication strategies. We should also point out the importance of devices such as specifications or the information exchange groups set up within cooperatives as part of the cognitive and organisational learning processes which enable changes in practice. In France and elsewhere, at a time when various initiatives are being developed to market pork or chicken as being “raised without antibiotics”, the technical-economic framework provided by cooperatives and the health framework provided by veterinarians constitute an important lever for coming to the decision to learn how to reduce antibiotic use.

“Obviously, every time there are new specifications or changes on the farm, practices change too. From a work standpoint (…) maybe it’s still too soon for us to fully master these new production methods, but we’ll see how it goes as we gain experience. With the advice we get from the technicians, we adjust the feed, we adjust the portions, we observe and we monitor. That’s also the role of the CETA (“Centre d’études techniques agricoles” (technical agricultural study centre). Information exchange groups created in the 1960s, which have now often been recreated within cooperatives.), we compare our results in the CETA, we look at the way everyone works and then we make an assessment.”

[Farmer]

## 4. Conclusions

Although the studied trajectories of change are far from homogeneous and whilst we can confirm that there is no “one best way” of reducing the use of antibiotics in animal farming, we have identified several dimensions that are necessary to the antibiotic reduction process (three learning processes and the role of social networks). This perspective allows us to think anew about farmer’s motivations and perceptions, by reintegrating them on the one hand into a longer temporality and on the other hand into a broader system of professional relations. The results have shown us the importance of triggering factors, such as health-related events or changes in networks or of economic and technical objectives which shape the trajectories of change. The role of actors who provide farmers with diverse resources in terms of methods and knowledge is also central. So whilst veterinarians still have a vital place in the implementation of demedication strategies, they are often linked to other important actors, such as technicians or other farmers. This result is important for policy recommendations because it shows that the reduction of antibiotic use in livestock is not the responsibility of the sole farmer or veterinarian. The fight against antimicrobial resistance does not only rely on technical solutions (*i.e.*, alternatives to antibiotics) but should be based on a global strategy aiming at changing the whole farming system, including the organization of work, animal health representation or the social image of the livestock industry.

Finally, this study has brought to light contextual factors and broader collective dynamics which need to be studied in greater depth at a future date. This is especially the case with cooperatives’ commercial strategies, with the introduction of specifications which encourage reduced used of antibiotics. Overall, whilst the study uses a panel of pig farms chosen from among those which had most significantly reduced their consumption of antibiotics, the results highlight a set of levers (three levels of learning through which the effects of technical, economic, social and organisational factors are combined) which can, *a priori*, be applied in other farms or other sectors of production. Nevertheless, we still need to know more about the barriers that can prevent the reduction of antibiotic use in livestock production. Complementary studies will have to be carried out on farmers that still fail to reduce their consumption of antibiotics, especially considering that farming systems and veterinary drug regulations could differ from one country to another. Indeed, the French context has some particularities that need to be taken into account in order to compare it with different countries (importance of family farming, “coupling” of prescription and dispensing of veterinary drugs, *etc.*). However, such an international (and qualitative) comparison could enlighten new aspects of trajectory of change that will eventually be helpful and provide keys for all animal health actors, and help them reach their objectives in terms of reduced antibiotic use in animal farming.
